# Ethnic differences in patients’ perceptions towards isolated orthopedic injuries: a pilot study

**DOI:** 10.1186/s40814-017-0188-x

**Published:** 2017-11-07

**Authors:** Boris A. Zelle, Gurpreet Singh, Deanna L. Kitchen, Roberto J. Fajardo, Mohit Bhandari, Melissa A. Valerio

**Affiliations:** 1 Department of Orthopaedics, UT Health San Antonio, 7703 Floyd Curl Dr, MC-7774, San Antonio, TX 78229 USA; 2 Division of Orthopaedic Surgery, McMaster University, 293 Wellington Street North, Suite 110, Hamilton, ON L8L 2X2 Canada; 3 UT Health School of Public Health, San Antonio Regional Campus, 7411 John Smith Drive, Suite 1100, San Antonio, TX 78229 USA

**Keywords:** Healthcare disparities, Perceptions, Injury, Hispanic

## Abstract

**Background:**

Patients’ perceptions of their healthcare have been reported to influence clinical outcomes following orthopedic trauma. Findings across clinical outcomes have demonstrated significant differences in perceptions towards healthcare between Hispanics and non-Hispanic whites. However, ethnic disparities in perceptions towards orthopedic injuries have not been examined in the literature.

**Aim of study:**

The aim of this pilot study is to explore whether Hispanic patients with isolated orthopedic injuries will demonstrate different perceptions towards their injury as compared to non-Hispanic white patients. The pilot data will be used to inform a subsequent larger clinical investigation and interventional study.

**Methods:**

A total of 43 patients (31 Hispanics and 12 non-Hispanic whites) with isolated orthopedic injuries requiring surgical treatment were enrolled in this cross-sectional observational pilot study. Outcome measures included the Questionnaire of Perceived Injustice (QPI), Short-Form 36 Health Survey (SF-36v2), Pain Catastrophizing Scale, and Consumer Assessment of Healthcare Providers and Systems (CAHPS) Cultural Competence (CC) item set.

**Results:**

The CAHPS was completed by 34 patients, and the remaining scoring systems were completed by all 43 subjects enrolled in this study. Hispanic patients trended towards higher QPI scores indicating poorer outcomes than non-Hispanic whites (mean difference [MD] 5.4, 95%; confidence interval [CI] − 4.4, 15.2). The mental component summary score of the SF-36 trended lower in Hispanics as compared to non-Hispanic white (MD − 6.8, 95%; CI − 15.0, 1.4). Hispanic patients also expressed less trust in their doctor on a scale from 0 to 10 (MD − 1.0, 95%; CI − 1.9, − 0.1).

**Conclusions:**

Our study suggests ethnic differences in patients’ perceptions towards isolated orthopedic injuries. These results must be interpreted cautiously given the limited number of subjects in this pilot examination. We collected sufficient data to allow a sample size calculation for a subsequent larger clinical investigation. Future clinical investigations may determine the influence of ethnic differences in patients’ perceptions towards orthopedic injuries, identify their impact on the functional outcomes, and establish intervention strategies.

## Background

Patients’ perceptions of care have been reported to influence functional clinical outcomes following orthopedic trauma. Patients’ belief systems have been suggested to predict the recovery after whiplash injuries, back pain, hand injuries, tibia fractures, and mangled lower extremity injuries [1–5]. Although the impact of patients’ perceptions on the functional recovery after orthopedic trauma seems to be widely accepted, the topic has received limited attention in the orthopedic literature. Detailed knowledge of patients’ beliefs towards their orthopedic injuries appears crucial for understanding of disparities in clinical outcomes. Increased understanding of factors that influence outcomes may allow orthopedic surgeons to establish protocols for recognizing and more appropriately addressing patients’ perceptions to improve the functional clinical outcomes following orthopedic trauma.

It is well documented that various ethnic and racial disparities are associated with patients’ perceptions towards their healthcare [6–9]. For example, Hispanic patients appear to be less willing to participate in cancer screening due to mistrust of healthcare providers, fear of “being a guinea pig,” and fear of embarrassment [6]. In addition, Hispanic immigrants may be more likely to use alternative medicine as a first line of care and feel that they have less control over their own health, fatalism [7]. A recently published study recorded that fatalistic attitudes and medical system mistrust were more prevalent among minority men [10]. Furthermore, these attributes were associated with poorer physical and emotional well-being. This study data offers some insight into Hispanic perceptions of healthcare in general. However, these data cannot be extrapolated to examine the barriers and facilitators for orthopedic trauma patients. Anecdotal reports have also suggested that Hispanic patients with musculoskeletal injuries have a higher prevalence of posttraumatic stress disorder symptoms than non-Hispanic whites [9]. However, the underlying factors contributing to this health disparity remain unclear.

Given Hispanics represent the fastest growing ethnic population in the USA, increasing by 15.2 million (43%) between 2000 and 2010. By 2050, the Hispanic population is expected to almost double in size from 16 to 30% of the entire USA population [11]. These growth trends emphasize the urgent need for detailed knowledge on characteristics of the Hispanic patient population.

This pilot study compared patients’ perceptions towards isolated orthopedic injuries in a sample of Hispanic and non-Hispanic white patients. The purpose of this pilot study is to explore whether Hispanic patients with isolated orthopedic injuries will demonstrate different perceptions towards their injury as compared to non-Hispanic white patients.

## Methods

The study was performed at a university-based level-1 trauma center in San Antonio, Texas. The population within the city of San Antonio includes approximately 60% Hispanics and approximately 30% non-Hispanic whites (https://www.sanantonio.gov). The protocol was approved by the Institutional Review Board (IRB) of the University of Texas Health Science Center at San Antonio. A total of 43 consecutive patients (31 Hispanics and 12 non-Hispanic whites) were enrolled in this cross-sectional observational study. Patients were identified by their treating physician at the orthopedic trauma clinic at our university-based level-1 trauma center. Patients who met the following inclusion criteria were eligible for participation in this study: (1) had an isolated orthopedic injury requiring surgical fracture treatment, (2) six-week follow-up visit following their most recent surgical fracture treatment, (3) between the age of 18 and 65 years, (4) willing to provide informed consent, and (5) self-reported ethnicity of Hispanic or non-Hispanic white. The following patients were excluded from the study: (1) patients with significant non-orthopedic injuries (abbreviated injury scale [12] > 2), (2) patients with more than one orthopedic injury requiring surgical fracture treatment, (3) a history of mental illness, (4) decisional impairment, or (5) unable to read and/or write either English or Spanish.

### Patient recruitment and data collection

Potential subjects were approached about their study participation when they returned to our trauma clinic for their six-week follow-up appointment following a surgical fracture treatment. Written informed consent was obtained from each patient participating in this study prior to data collection. Patients were offered to choose between the English or the Spanish version of the study questionnaire. Patients were handed the questionnaire and asked to (1) complete the survey on site or (2) submit using a stamped return envelope. Upon receipt of the completed study questionnaire, patients received a $40 gift card for their study participation. Demographic and clinical data were obtained from the electronic medical record including demographic information: patient date of birth, date of injury, diagnosis of injury, American Society of Anesthesiologists’ (ASA) physical status classification [13], and type of surgical treatment rendered. Protected health information (PHI) was handled confidentially and according to approved institutional and study protocol guidelines.

### Study questionnaire

The study questionnaire was a compilation of valid and reliable scoring systems including the Questionnaire of Perceived Injustice (QPI), a wording modification of the Injustice Experience Questionnaire [14, 15], Short-Form 36 Health Survey (SF-36v2) [16], the Pain Catastrophizing Scale (PCS) [17], and Consumer Assessment of Healthcare Providers and Systems (CAHPS) Cultural Competence (CC) item set [18]. Additional questions on the patient’s self-reported ethnicity, native language, country of birth, and social history (smoking, alcohol, drug abuse, educational level, income level) were included in the study questionnaire.

The Injustice Experience Questionnaire is a 12-item scoring system that has been validated and used in both the English and Spanish language [14, 15]. A minimal important difference (MID) has not been defined for this outcome measure. The SF-36v2 is a standardized scoring system that has been used in numerous clinical trials [16]. It is divided into two main categories, physical component summary score and the mental component summary score. It can also be subdivided into eight subscales. The Spanish version of the SF-36v2 has been validated and has been widely used in clinical studies [19, 20]. A MID of 3 has been identified for the physical and mental component summary scores of the SF-36 [21, 22]. The PCS is a 13-item questionnaire that has been validated in the English and Spanish language [17]. To our best knowledge, the MID has not been determined for the PCS.

The CC item set of the CAHPS is a validated system that has been validated in the English and Spanish language [18]. It allows for calculation of two composites: (1) Providers are caring and inspire trust (five items), and (2) Providers are polite and considerate (three Items). It also includes a Likert scale from 0 to 10 for trust in the healthcare provider. The composite of “provider caring and inspiring trust” includes the following five items: (1) In the last 12 months, did you feel you could tell this provider anything, even things that you might not tell anyone else? (2) In the last 12 months, did you feel you could trust this provider with your medical care? (3) In the last 12 months, did you feel that this provider always told you the truth about your health, even if there was bad news? (4) In the last 12 months, did you feel this provider cared as much as you do about your health? and (5) In the last 12 months, did you feel this provider really cared about you as a person? The composite of “providers polite and considerate” consists of the following three items: (1) In the last 12 months, how often did this provider talk too fast when talking with you? (2) In the last 12 months, how often did this provider use a condescending, sarcastic, or rude tone or manner with you? and (3) In the last 12 months, how often did this provider interrupt you when you were talking? The response options for each of these items include “never”, “sometimes”, “usually”, and “always”. The items were scored using the top box score method which calculates the rate of respondents who chose the most favorable response, e.g., “In the last 12 months, how often did this provider use a condescending, sarcastic, or rude tone or manner with you?” (most favorable response: never); “In the last 12 months, did you feel you could trust this provider with your medical care?” (most favorable response: always). The score for a composite represents the rate of most favorable responses for all items within the respective composite.

### Statistics

All statistical analysis was performed using Stata 14 (StataCorp, College Station, TX). Patients were divided into Hispanics versus non-Hispanic whites according to their self-reported ethnicity. No sample size calculation had been performed for the purpose of this pilot study as no comparable data was available in the current literature. The chi-square test was used to compare gender and smoking, between Hispanics and non-Hispanic whites. The Fisher’s exact test was used to compare the educational level and income between the two groups. The *T* test was used to compare age. Based on the results of this pilot investigation, a power analysis was performed in order to calculate how many patients would have to be included in a potential future trial in order to have 80% power to find a difference at the level of *p* = 0.05 for the QPI.

## Results

A summary of the demographic data is provided in Table 1. The isolated orthopedic injuries in these patients included various anatomic regions including injuries to the distal humerus, both bone forearm, distal radius, acetabulum, pelvic ring, hip, femoral shaft, distal femur, patella, tibial plateau, tibial shaft, pilon, ankle, calcaneus, and midfoot. No differences by demographic or clinical characteristics between the two groups were found.

**Table 1:** Demographic variable comparing Hispanic versus non-Hispanic white patients

	Hispanics (*n* = 31)	Non-Hispanic whites (*n* = 12)	Total (*n* = 43)	*p*
Age in years, mean (SD)	41.9 (12.4)	44.7 (15.3)	42.7 (13.1)	0.58
Gender (% female)	51.6% (16)	66.7% (8)	55.8% (24)	0.38
Smoking history % (*n*)	19.4% (6)	41.7% (5)	25.6% (11)	0.13
Annual income level % (*n*)^a^				
Less than $20,000	44.8% (13)	16.7% (2)	35% (14)	0.09
$20,000–$49,000	37.9% (11)	41.6% (5)	40% (16)	
$50,000–$79,000	13.8% (4)	25% (3)	17.5% (7)	
$100,000–$299,000	3.5% (1)	16.7% (2)	5% (3)	
Educational level % (*n*)				
8th grade or less	16.1% (5)	0% (0)	11.6% (5)	0.30
Some high school but did not graduate	9.7% (3)	0% (0)	7.0% (3)	
High school graduate or GED	38.7% (12)	50% (6)	41.9% (18)	
Some college or 2-year degree	29% (9)	25% (3)	27.9% (12)	
Four-year college graduate	3.2% (1)	8.3% (1)	4.7% (2)	
More than 4-year college degree	3.2% (1)	16.7% (2)	7.0% (3)	
ASA Class % (*n*)				
Class I	12.9% (4)	8.3% (1)	11.6% (5)	0.10
Class II	51.6% (16)	75% (9)	58.1% (25)	
Class III	35.5% (11)	8.3% (1)	27.9% (12)	
Class IV	0% (0)	8.3% (1)	2.3% (1)	

*SD* Standard deviation, *ASA* American Society of Anesthesiologists

^a^Annual income level available for total *n* = 41

The QPI, SF-36, and PCS were completed by all 43 patients enrolled in this study (Table 2). The QPI was found to point towards worse outcomes in Hispanic versus non-Hispanic white patients (mean difference [MD] 5.4, 95%; confidence interval [CI] − 4.4, 15.2). Hispanic patients showed a trend towards better physical component summary scores of the SF-36 (MD 4.7, 95%; CI 0.2, 9.3), while the mental component summary score of the SF-36 trended lower in Hispanics patients (MD − 6.8, 95%; CI − 15.0, 1.4). The magnitude of the differences for the SF-36 summary scores was greater than the MID of 3. The PCS did not show any trends between Hispanic patients.

**Table 2:** Outcome scores comparing Hispanics versus non-Hispanic whites

	Hispanics, mean (SD)	Non-Hispanic whites, mean (SD)	MD (95% CI)
SF-36			
Physical function	33 (32.0)	25.8 (27.2)	7.2 (− 14.1,28.4)
Role-physical	23.1 (29.0)	13.5 (14.3)	9.6 (− 4.1,23.2)
Bodily pain	35.8 (21.9)	36.4 (16.2)	− 0.7 (− 13.2,11.9)
General health	68.2 (21.9)	64.8 (13.7)	3.5 (− 8.0,14.9)
Vitality scale	50.4 (24.0)	48.4 (22.3)	2.0 (−14.2,18.1)
Social functioning	37.1 (30.2)	52.1 (27.6)	− 15.0 (− 35.1,5.1)
Role-emotional	55.0 (38.9)	57.6 (34.2)	− 2.6 (− 27.8,22.5)
Mental health	62.0 (22.7)	76.7 (16.6)	− 14.7 (− 27.6,− 1.7)
Physical component summary	34.9 (7.1)	30.2 (6.2)	4.7 (0.2,9.3)
Mental component summary	44.0 (13.0)	50.8 (10.9)	− 6.8 (− 15.0,1.4)
QPI	22.7 (12.0)	17.3 (14.3)	5.4 (− 4.4,15.2)
PCS	14.8 (12.9)	14.7 (12.1)	0.1 (− 8.6,8.8)
Trust with doctor (0–10)	8.9 (0.4)	9.9 (2.1)	− 1.0 (− 1.9,− 0.1)

*SD* standard deviation, *MD* mean difference, *CI* confidence interval, *SF-36* Short-Form 36 Health Survey, *QPI* Questionnaire of Perceived Injustice, *PCS* Pain Catastrophizing Scale

The CC item set of the CAHPS was completed by a total of 34 patients (27 Hispanics and 7 non-Hispanic whites). This was attributed to the length of the questionnaire and the wording of some questions that allowed skipping certain items. Hispanic patients seemed less likely to choose the top box score within the composite of “providers caring and inspiring trust” as compared to non-Hispanic whites, 64.8 versus 80% (odds ratio [OR] 2.21, 95%; CI 0.90, 5.43). No trends were observed for the composite score of “providers polite and considerate” with top box scores in 79% of Hispanics versus 81% in non-Hispanics whites (OR 1.13, 95%; CI 0.34, 3.80). In addition, Hispanic patients indicated less trust with their doctor on a scale from 0 to 10 (MD − 1.0, 95%; CI − 1.9, − 0.1).

A power analysis was performed for sample size estimation based on our pilot data. The QPI as our primary outcome was used for sample size estimation. The power analysis determined that 87 subjects would be needed in each arm to have an 80% chance of detecting a statistically significant difference between the QPI scores of Hispanics vs. non-Hispanic whites at the level of *p* = 0.05.

The following parametric formula was used to calculate the sample size:

*n* = 2 ([*α* + *β*]^2^[*σ*]^2^) / (*μ*_1_-*μ*_2_)^2^.

*α* = 1.96 (*z*-score for a two-sided test with an alpha of 0.05).

*β* = .84 (*z*-score for 80% power).

*σ* = 12.66 (calculated from pooled standard deviation formula using the means, standard deviations, and sample sizes for the QPI. The pooled version of the formula was used since the Hispanic and non-Hispanic groups had different sample sizes).

*μ*_1_ = 22.7 (the mean QPI score for the Hispanic group).

*μ*_2_ = 17.3 (the mean QPI score for the non-Hispanic group).

## Discussion

It has been widely suggested that patients’ perceptions may have a significant influence on functional recovery following orthopedic injuries [1–5]. While in other areas of medicine, ethnic and racial disparities with regard to patients’ perceptions towards their healthcare have been observed, patient perceptions have not been a focus of research in the orthopedic literature. In this pilot study, we aimed to collect data to identify whether ethnic differences in patients’ perceptions towards isolated orthopedic injuries should be further examined. Among Hispanic patients with isolated orthopedic injuries, we identified a trend towards slower mental recovery, greater perceived injustice, and less trust in the healthcare provider despite a favorable physical recovery. We believe that the study methodology and outcome measures used to explore the impact on clinical outcomes were shown to be feasible for larger clinical investigations. The QPI, SF-36, and PCS demonstrated a high rate of completion. We also conclude that patients may require assistance when completing the CAHPS.

Our study has both strengths and limitations. We chose a patient population that was homogenous with regard to the diagnosis of an isolated orthopedic injury requiring surgical treatment. However, we included a broad spectrum of orthopedic injuries that included various injured anatomic areas. Future investigations may focus on one particular type of injury in order to further improve the homogeneity of the study population. We believe that, overall, the outcome measures used in this study were appropriate and useful to answer the study question. We would like to acknowledge that the choice of outcome measures was somewhat limited by the fact that we needed to use questionnaires that had been validated in both the English and Spanish language. This precluded us from the use of other interesting outcome tools, such as the Somatic Pre-Occupation and Coping (SPOC) questionnaire [2]. Future studies investigating healthcare disparities in other patient populations may tailor their choice of outcome measures according to the language requirements within their respective patient population. We also would like to acknowledge that our study is a single time-point study. The six-week follow-up time point appeared appropriate as it was felt that the patients will be out of the acute pain phase at that time. Future proposals need to include longitudinal study designs that will allow for correlating patient perceptions with long-term functional outcomes after orthopedic injuries. We, therefore, suggest that this pilot study lays the ground for subsequent larger prospective clinical investigations. We do not have a robust justification for the sample size of this study. We used a convenience sample based on recommendations from the literature suggesting that this number is large enough for a pilot study [23]. Yet, the sample size of this pilot study was too small to make adjustments for potentially confounding demographic and clinical variables, such as age, gender, educational level, income level, employment status, insurance status, smoking, alcohol, drug abuse, medical comorbidities, and types of injuries. However, subsequent larger investigations need to include logistic regression models in order to make adjustments for these potentially confounding variables. Despite the relatively small sample size, we were able to identify fairly remarkable trends and differences between Hispanics and non-Hispanic whites. However, the results of our study must be interpreted with caution and may need to be confirmed in subsequent larger clinical investigations. Finally, our study focused on differences between Hispanics and non-Hispanic whites and we cannot make any conclusions about other minority groups. Future studies may extend this study question to other ethnic and racial groups.

To our best knowledge, the question of ethnic differences in patients’ perceptions towards isolated orthopedic injuries is novel within the literature. Therefore, the results recorded in this pilot study are difficult to compare to published literature. Anecdotal reports have suggested a higher rate of posttraumatic stress disorder among Hispanic polytrauma patients as compared to non-Hispanic whites [9]. With regard to perceptions towards healthcare, we are only aware of data from other areas of medicine [6–8]. These investigations have shown for instance that Hispanic patients are less likely to participate in cancer screening due to mistrust, are more likely to report fear of “being a guinea pig,” are more likely to report fear of embarrassment, more frequently use alternative medicine, and feel that they are less in control over their own health. However, these data cannot be extrapolated to the orthopedic patient population and further clinical investigations seem necessary.

## Conclusions

Our pilot study showed that the methods of data collection are feasible for our clinical setting. The study questionnaire showed a satisfactory completion rate in both Hispanic and non-Hispanic white patients. Our study suggests that ethnic differences in patients’ perceptions towards isolated orthopedic injuries whereby Hispanic patients may have a slower mental recovery, may be more likely to express perceived injustice, and may express more mistrust towards their provider than non-Hispanic whites. These results must be interpreted cautiously given the limited number of subjects in this pilot examination. However, our study provides a basis for future clinical investigations. We collected sufficient data to allow a sample size calculation for a subsequent larger clinical investigation. Future clinical investigations may determine the influence of ethnic differences in patients’ perceptions towards orthopedic injuries, identify their impact on the functional outcomes, and establish appropriate patient and provider intervention strategies.

## Abbreviations

ASA: American Society of Anesthesiologists
CAHPS: Consumer Assessment of Healthcare Providers and Systems
CC: Cultural Competence
CI: Confidence interval
IRB: Institutional review board
MD: Mean difference
MID: Minimal important difference
OR: Odds ratio
PCS: Pain Catastrophizing Scale
PHI: Protected health information
QPI: Questionnaire of Perceived Injustice
SF-36: Short-Form 36 Health Survey
SPOC: Somatic Pre-Occupation and Coping
